# Influence of pig farming on human Gut Microbiota: role of airborne microbial communities

**DOI:** 10.1080/19490976.2021.1927634

**Published:** 2021-06-01

**Authors:** Julia Moor, Tsering Wüthrich, Suzanne Aebi, Nadezda Mostacci, Gudrun Overesch, Anne Oppliger, Markus Hilty

**Affiliations:** a Institute for Infectious Diseases, University of Bern, Bern, Switzerland; b Graduate School of Cellular and Biomedical Sciences, University of Bern, Bern, Switzerland; c Institute of Veterinary Bacteriology, University of Bern, Bern, Switzerland; d Unisante, Department of Occupational and Environmental Health, University of Lausanne, Lausanne, Switzerland

**Keywords:** Occupational exposure, pig farming, one health, human stool, pig, bioaerosol, microbial communities

## Abstract

It has been hypothesized that both genetics and diet influence the composition of the human cecal microbiota. However, it remains unclear whether and how occupational exposure to microbes impacts the microbial communities in human guts. Using a One Health approach, we visited pig farms (n = 26) and collected stool specimens from pig workers (n = 59), pig barn air samples (n = 19), and rectal swabs from pigs at three different growth stages (n = 144). Stool samples from cattle workers were included as a control group (n = 22). Each sample’s microbiota was characterized using 16S rRNA gene sequencing and the DADA2 pipeline.

We obtained a significantly different clustering of the microbial compositions of pig and cattle workers by permutational multivariate analysis of variance (PERMANOVA; *P* < .001). Workers primarily exposed to pigs had higher relative abundances of *Prevotellaceae* and less *Bacteroidaceae* than workers exposed to cattle. We also found that the microbial compositions of pig workers’ stool samples shared extensive fractions with the samples from their pigs. We also identified amplicon sequencing variants (ASVs) in the airborne microbiota which were likely involved in zoonotic transmission events.

We hypothesize that ASVs originating from pig feces are aerosolized and, through breathing, get trapped in the pig farm workers’ upper respiratory tract from where they can get swallowed. Consequently, some of the animal associated ASVs are transferred into the gastrointestinal tracts (GITs) which leads to changes in the composition of the human gut microbiota. The importance of this finding for human health must be investigated further.

## Introduction

The human gut is the ecological niche of a vast number of bacteria whose composition has been well studied.^1^ It has been hypothesized that the composition of the gut’s microbiota is shaped by the host’s genetics and such important environmental factors as diet and geography.^2–4^ The use of antimicrobial drugs has been associated with shifts in the composition of gut microbiota.^5^ However, these effects differ in strength and duration, depending on the type of antimicrobial drugs used.^5^ Conversely, traveling does not seem to be associated with lasting changes in the gut’s microbiota. Recent studies have reported that the region visited, the type or duration of travel, and the use or type of malaria prophylaxis had no significant influence on the composition of gut microbiota.^6,7^

In general, little is known about whether exposure to chemical substances or bioaerosols is linked to a changing composition of the gut microbiota, thereby impacting human health. A recent review suggested that occupational exposure to airborne engineered nanomaterials could lead to significant interactions with the gastrointestinal tract and affect its microbiota.^8^ Another study showed that patients who suffered from silicosis after occupational exposure to silica presented with a dysbiosis of the gut microbiota. However, due to its observational design, the study was unable to draw conclusions about the causation of this association.^9^ Moreover, animal studies have demonstrated that some chemicals used in occupational settings (e.g., various pesticides and metals) can induce changes in the microbiome.^10,11^

In addition, studies have been done investigating the influence of occupational exposure to pigs on human nasal microbiota. One of those studies analyzing the microbiota in the nares of pigs and pig workers showed that exposure to pig farm environments was strongly associated with specific microbial signatures (including alpha- and beta-diversity), which were reflected in the microbiota of the human nose.^12^ The study also investigated the air-transmissible microbiota and suggested that animal–human transmission of bacteria occurred through the air in pig farms.^12^ In a follow-up study it has then been shown, that bacteria present in pigs’ noses are more commonly identified in pig workers’ microbiota in winter than in summer.^13^ Taken together, these results strongly indicate that pig farming is associated with a distinct human nose microbiota.^12^ In addition, a very recent study has hypothesized that occupational contact between workers and pigs might also result in a bacterial community shift in the human gut.^14^ However, there is still a lack of knowledge as to if and how occupational microbial exposure is linked to changes in the human gut’s microbiota.

The present study aimed to: (i) describe the potential influence of pig farming on the human cecal microbiota; (ii) define the shared sequence variants (ASVs) derived from the 16S rDNA sequencing of pig rectal swabs, air samples collected in pig barns, and pig workers’ stools; and (iii) identify if there is a pig farm-specific microbiota in humans, pigs and air.

## Results

### Characterization of sampling population and sequence analysis

**Figure 1.**
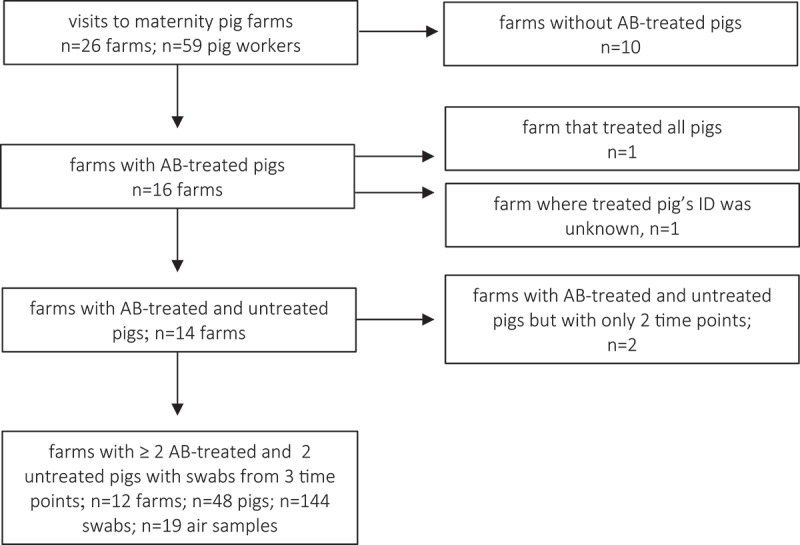
Flow chart of pig farm selection and the number of samples from pigs, pig workers, and air

In total, 26 pig farms were visited and the stool samples of 59 pig workers were collected. A subset of 12 farms was chosen for the sequencing of animal and air samples because they had both untreated pigs and pigs that had been exposed to antimicrobials during suckling or weaning but not fattening (Figure 1). Specifically, the rectal swabs of two antimicrobial-treated and two untreated pigs per farm were taken during their three different growth stages (suckling, weaning, and fattening) (n = 48 animals; n = 144 samples). From these 12 farms, barn air samples (n = 19) were collected from either the weaning or fattening units (Figure 1 and Supplementary Table 1). Self-collected stool samples from a control group of cattle workers were also incorporated to assess pig farming’s effects on human cecal microbiota (n = 22). All samples were processed to enable subsequent 16S rRNA microbiota analysis. An overview of the number of samples sequenced from animals, humans, and barn air can be found in Figure 1. In total, the study included 81 human, 144 animal, and 19 air samples, with a total of 27,393,755 reads. On average, we received 112,269.5 (SD = 44,641) sequencing reads per sample which were subsequently clustered into 12,393 ASVs overall (Supplementary Table 1).

### Alpha-diversity values and changes to the composition of the microbial community in human cecal samples

**Figure 2.**
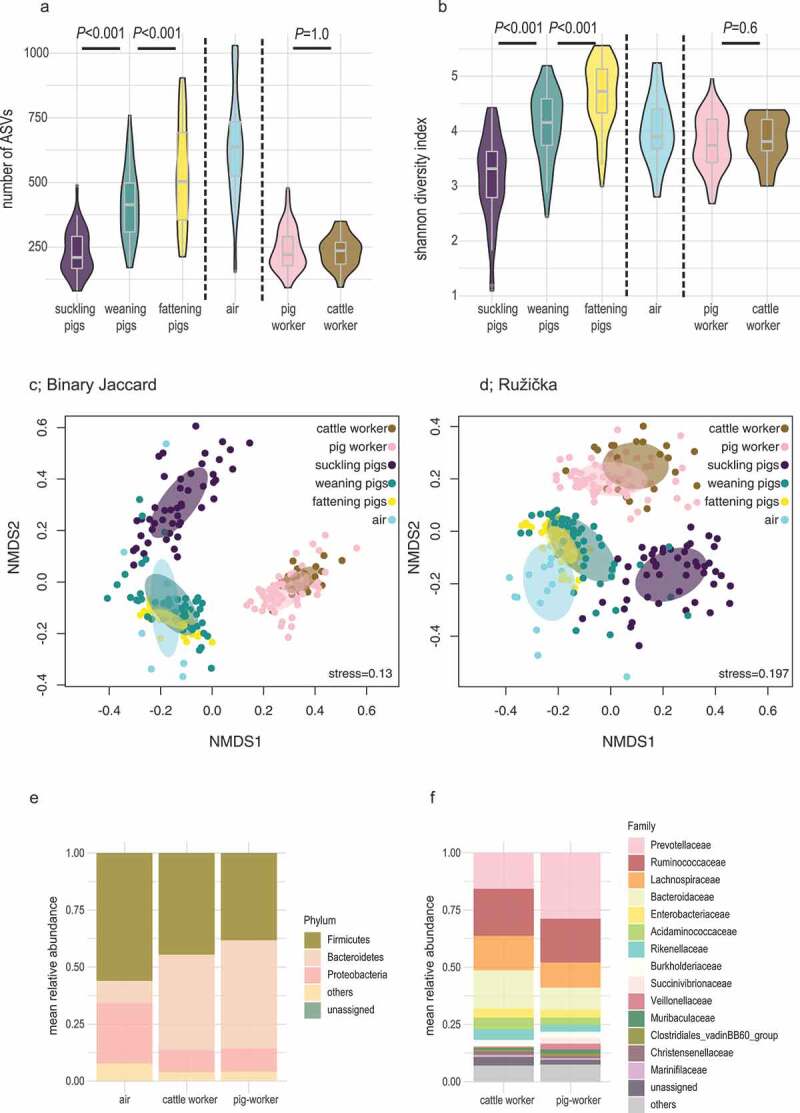
Alpha- and beta-diversity analyses of samples from pigs, air, pig workers and cattle workers

As for alpha-diversity analyses, we first counted the number of ASVs (Richness; Figure 2a) and calculated the Shannon diversity indices (SDI) for each sample (SDI; Figure 2b). We found that values of both indices increased with pig growth stages, reaching their highest values in fattening pigs. Over all the samples, richness was highest in air samples, but SDI values were not. There were no significant differences between the alpha-diversity values of stool samples from cattle workers and pig workers (Figure 2a and 2b).

Pigs were sampled at three different growth stages, as suckling pigs, weaning pigs, and fattening pigs. To illustrate alpha-diversity, (A) shows the values for richness (observed ASVs) and (B) shows the Shannon diversity indices, both based on sample types. To illustrate beta-diversity, (C) shows the unweighted (Jaccard) and (D) shows the weighted (Ružička) distances in the samples’ microbiota compositions (reduced to a two-dimensional space by NMDS); the 95% confidence ellipses for the group centroids are also shown. (E) illustrates the relative abundance of phyla and (F) the relative abundance of families among air, cattle workers and pig workers.

Next, we calculated the binary and abundance-based distance matrices and created ordination methods-based non-metric multidimensional scaling (NMDS) plots (Figure 2c and 2d). For both types of calculations, we found separate clusterings of suckling pigs and of fattening and weaning pigs, whereas air samples were very closely clustered with the latter. The human samples were clearly different from the pig and air samples, and they segregated into two slightly overlapping clusters of pig workers and cattle workers. However, performing PERMANOVA using exclusively human data showed significant separation of the pig and cattle worker using both, the binary (PERMANOVA; F-value = 2.2, *P* < .001) and abundance-based distance matrices as input data (PERMANOVA; F-value = 1.7, *P* < .001). In addition, the 95% confidence ellipse for the group centroid of pig workers showed a closer proximity to weaning and fattening pigs as compared to cattle workers indicating that pig farming may affect the composition of the microbial communities in human guts (Figure 2c and 2d).

Focusing on the phyla distribution, the mean Bacteroidetes/Firmicutes ratio found between the two human groups was different showing values of 1.25 for pig worker and 0.96 for cattle worker (Figure 2e). Within the phylum Bacteroidetes, the family *Prevotellaceae* showed higher relative abundance in pig workers than in cattle workers (Wilcox-test; *P*_Crude_ = 0.007; *P*_corrected_ = 0.07), whereas the opposite was observed for the *Bacteroidaceae* (Wilcox-test; *P*_Crude_ = 0.03; *P*_corrected_ = 0.3; figure 2f).

### Alpha- and beta-diversity values of stratified animal and human samples

**Figure 3.**
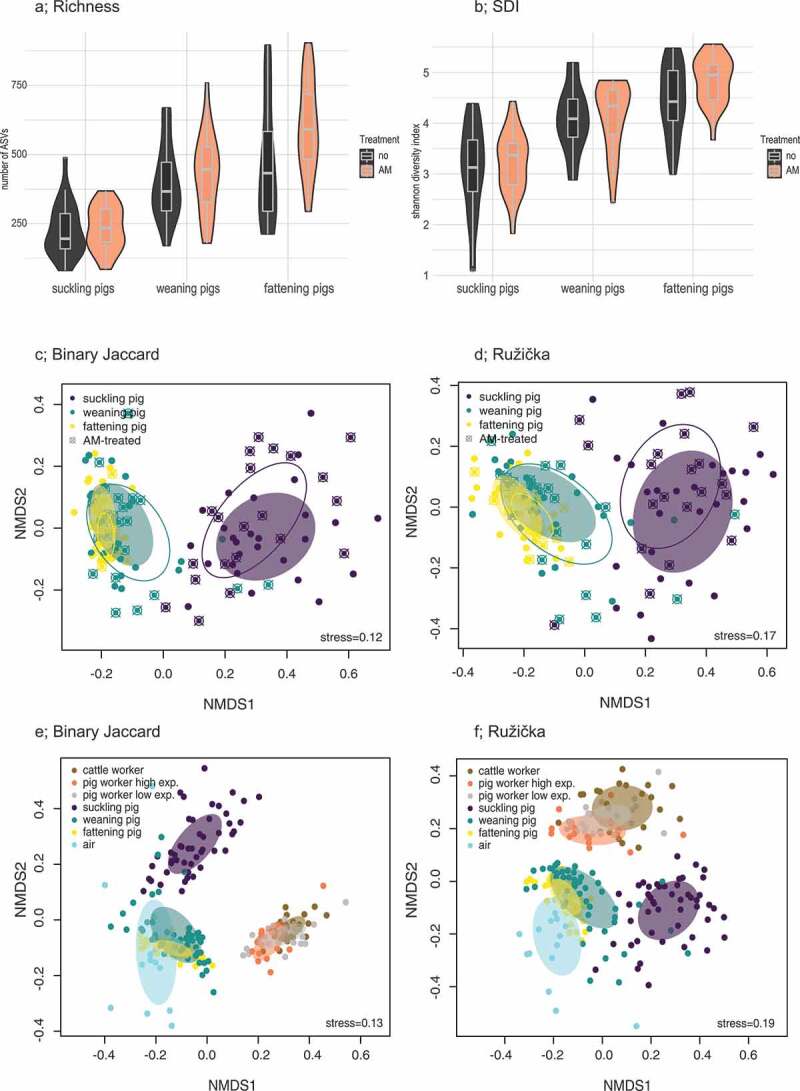
Alpha- and beta-diversity analyses of stratified animal and human samples

The animal samples were next stratified according to their antimicrobial treatment status. Again, the numbers of ASVs were counted (Richness; Figure 3a) and the Shannon diversity indices (SDI) were calculated (SDI; Figure 3b). Alpha-diversity values did not differ significantly between the treated and untreated samples. Likewise, the NMDS plots displayed no clustering by treatment status in any growth stage (Figure 3c and 3d). The types of antimicrobial drugs and treatment timepoints were diverse and can be found in Supplementary Table 2.

(A) shows the values for richness (observed ASVs) and (B) shows the Shannon diversity indices based on pig growth stage (suckling, weaning, and fattening pigs) and antimicrobial (AM) treatment. For the beta-diversity, (C) illustrates the unweighted (Jaccard) and (D) illustrates the weighted (Ružička) distances in the microbiota composition for the same stratified animal samples (reduced to a two-dimensional space by NMDS). (E) illustrates the unweighted (Jaccard) and (F) illustrates the weighted (Ružička) distances in the microbiota composition for pig workers with low exposure (≤ 2 h/day) and high exposure (usually full-time, ≥ 8 h/day).

Furthermore, we stratified the human samples according to pig workers with low exposure (≤ 2 h/day) and high exposure (≥ 8 h/day) to pig barn environments. Notably, as indicated with the 95% confidence ellipses for the group centroids, highly exposed pig workers clustered further away from the cattle workers, and the less exposed pig workers settled in the middle of the two. This indicated a shift in microbial composition toward the pigs as exposure time increased using binary distance as well as abundance-based distance matrices (Figure 3e and 3f). Indeed, ANOSIM analyses showed a significant separation between highly exposed pig workers and cattle workers (*P* < .001); in contrast, this was not found between less exposed pig workers and cattle workers (Supplementary Table 3).

### *ASVs of* Prevotellaceae *were transmitted via the air*

**Figure 4.**
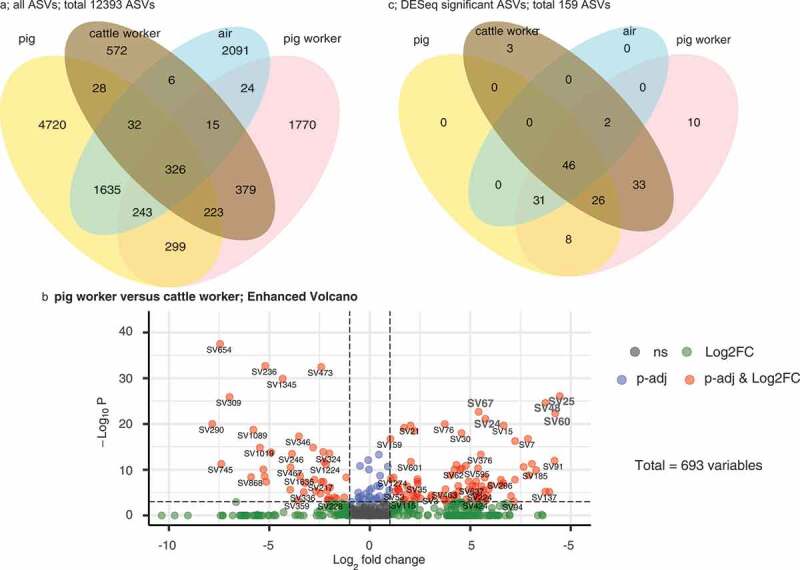
Venn diagrams and differential abundance of ASVs

We subsequently investigated which and how many ASVs were shared between the different sample types (Figure 4a). Overall, 3279 ASVs were found in pig worker but only 1581 in cattle worker samples. In addition, we found that a sizable number of ASVs (n = 1635) was shared between pigs and the air which may indicate that many of the ASVs from pig feces dispersed in aerosols. The taxonomic assignments of all the ASVs can be found in Supplementary Table 4. Next, we performed a differential abundance analysis for the ASVs of pig worker and cattle worker samples. Overall, 159 ASVs were identified in significantly higher numbers in either pig (n = 117 ASVs) or cattle workers (n = 42 ASVs; Figure 4b).

(A) Venn diagram showing the numbers of shared ASVs between cattle workers, pig workers, pigs, and air. (B) Differential abundance of ASVs in cattle workers and pig workers. DESeq analysis shows the fold changes of ASVs in bacterial communities of cattle workers versus pig workers. The negative Log2 fold changes indicate higher relative abundances of ASVs in cattle workers, whereas positive Log2 fold changes mean the opposite. The most significant ASVs are indicated in gray and their taxonomic assignment is mentioned in the text above. Overall, the Log2 fold changes of 159 ASVs were identified as being significantly higher in either pig or cattle farming. (C) Venn diagram showing the distribution of the 159 ASVs with significant negative or positive Log2 fold changes among cattle workers, pig workers, pigs, and air.

The most significant ASVs associated with pig workers were assigned to the genera *Prevotella* (ASV24, ASV25, ASV60 and ASV67) and *Acidaminococcus* (ASV48). As for cattle workers, the most significant ASVs belonged to the genera *Bacteroides* (ASV236 and ASV654) and *Phascolarctobacterium* (ASV309 and ASV473). Importantly, the differential abundance analyses mainly revealed ASVs of *Prevotellaceae* for the pig workers, mirroring the above received bacterial family-based findings (figure 2f).

Furthermore, we investigated whether the above mentioned 159 ASVs were also present in the air and pig samples (Figure 4c). Many of them were identified in pigs (n = 111) and barn air (n = 79). Moreover, 31 ASVs were found in pig workers, pigs, and pig barn air but not in cattle workers, indicating that they were zoonotically transmitted in pig farms. The relative abundances of these 159 ASVs are illustrated in supplementary Figure 1 as a heat map. Results indicated that ASV3 (*Megasphaera*), ASV7 (*Prevotella*), ASV15 (*Muribaculaceae*), ASV25 (*Prevotella*) and ASV30 (*Dialister*) had high relative abundances in pigs, pig workers, and air but not in the samples from cattle workers. Taken together, these analyses enabled us to identify the ASVs which were likely involved in the zoonotic transmission of microbiota.

### Farm-specific microbial composition of pig samples

**Figure 5.**
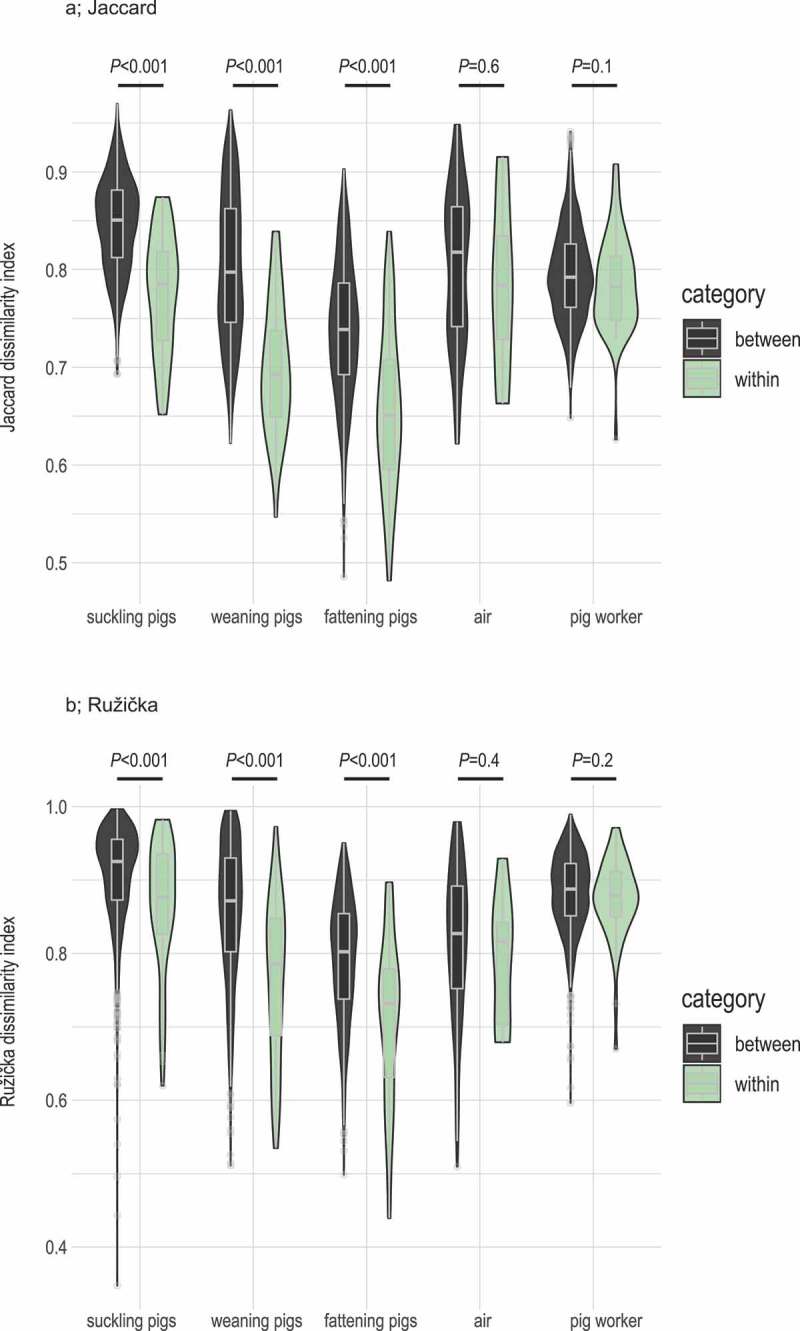
Within- and between-pig-farm dissimilarity analyses

Finally, we investigated whether the microbial communities identified were farm specific. To do this, we compared the values of the binary (Jaccard) and abundance-based (Ružička) distance matrices within farms (within beta diversity) to those between different farms (between beta diversity). We found that the values of both Jaccard and Ružička dissimilarity indices were significantly lower in pig samples at all three growth stages (suckling, weaning, and fattening) from ‘within farms’ than in ‘between farms’ (Figure 5a and 5b). In contrast, no differences were found for the air and stool samples from pig workers. Additional analyses of the dissimilarity index values of pig workers to pigs at the three growth stages and to air showed no significant within-between farm diversity effect (Supplementary Figures 2A and 2B). Taken together, this indicated that the microbiota among pigs were farm-specific, whereas air and pig workers’ microbiota were not. In other words, while we detected an overall effect of pig farming on the composition of human cecal microbiota composition, this effect cannot statistically be pinpointed to the individual pig farms.

(A) shows unweighted (Jaccard) and (B) shows weighted (Ružička) distance matrices values for ‘within-farm’ and ‘between-farm’ microbiota composition. Dissimilarity values were significantly higher for suckling, weaning, and fattening pigs ‘between farms’. No significant differences were found for air and pig workers’ stool samples.

## Discussion

This study made a cross-sectional characterization of the microbiota of pigs, air, and human workers. We found that the relative abundances of *Prevotellaceae* and *Bacteroidetes* were respectively higher and lower among pig workers than among cattle workers. More specifically, we identified ASVs of *Prevotellaceae* but also of other bacterial families that were simultaneously present in pigs, air, and pig workers, but not cattle workers. This indicates that aerosolization of pig feces bacteria was occurring, enabling the airborne transmission of zoonotic ASVs to pig workers.

In general, bacterial species of *Prevotellaceae* (*Prevotella* spp.) are common and important bacterial colonizers of human and porcine guts.^15,16^ It has been shown that if species of the genera *Prevotella* and *Bacteroides* co‑occur in the gut, one or the other predominates, suggesting antagonistic behavior.^16^ Indeed, we observed that greater relative abundances of *Prevotella* were associated with fewer *Bacteroides* in pig workers and vice-versa in cattle workers.

Greater numbers of *Prevotella* have been linked to vegetarianism in Western populations,^4^ but this type of diet is likely not characteristic of pig workers. Therefore, factors other than diet must be considered as potential explanations for greater proportions of *Prevotella* in pig workers. One explanation could be that the inhalation of airborne bacteria is associated with the ingestion of a portion of them. Indeed, it has been shown previously that a fraction of inhaled microorganisms is ingested as large particles (> 5 microns) are deposited in the upper respiratory tract and then swallowed.^17,18^ Based on available quantitative microbial risk assessment models,^19^ this fraction is considered to lie between 10%–80%.^19,20^ Applying this range of ingested microorganisms to the mean airborne concentration of bacteria found in the same Swiss pig farms in a previous study (3.6 x 10^9^ cfu/m^3^), ^21^ we estimated that a worker with a normal breathing rate ingested between 6.9 × 10^5^ cfu and 5.5 × 10^6^ cfu during 4 hours of work in a pig barn.

Another potential exposure route to zoonotic bacteria is direct contact with contaminated surfaces and the hand-to-mouth route. However, as numbers of ASVs shared by the three groups pigs, air, and pig workers (n = 31) were considerably higher than the number of shared ASVs by the two groups pigs and workers (n = 8), this exposure route seems to be less relevant for a corresponding alteration of the gut microbiota.

Overall, over 3000 ASVs were found in pig worker but only 1581 in cattle worker samples. As outlined above, we speculate that pig farmers incorporate ASVs from pig feces (via the air) and these ASVs can, therefore, be detected in addition to the core ASVs of the human gut. The fact that fewer ASVs were detectable in cattle worker samples indicate that no or only few ASVs from cattle feces are transmitted to humans though we did not analyze feces samples from cattle to prove this assumption. However, the finding may indicate that less ASVs from cattle feces are aerosolized, due to the type of the ASVs in the cattle feces or the manner the cattle are kept at the farm. Alternatively, it might also be explained by the differing bovine digestive systems. Whereas diet and the gastro intestinal tract of swine and humans are more alike (omnivores), the cattle mainly eat plant fibers which might explain facilitated uptake of ASVs from the former.

We also showed that the duration of exposure to pigs was an important factor in shaping the microbiota. This was observed in our sample groups since there was significant difference between cattle workers and the group of pig workers working ≥ 8 h/day, but not with the group working ≤ 2 h/day.

A very recent study examined the impact of pig farm environments on changes in the gut microbiome and resistome of Chinese veterinary students who had been occupationally exposed to them.^22^ Similarly, to the present study, it showed that farm exposure shaped students’ gut microbiomes, although different bacterial taxa were identified as being relevant in the transmissions. Specifically, moderately fewer *Bacteroidetes* and more *Proteobacteria* (especially *Gammaproteobacteria*) were observed.^22^ We hypothesize that these disparities arose from differing farming and management practices used in Chinese and Swiss pig farms. Further, Chinese and Swiss diet differ which has profound influence on the GIT microbiota.^2–4^

Furthermore, our findings indicated that the microbial compositions of pigs’ guts were specific to each farm. This was not a surprising finding as it has been shown that pig breeding, including feeding, has a profound effect on pig gut microbiota.^23,24^ It is surprising, however, that airborne microbial communities were not farm specific. One potential explanation for this could be that barn air is polluted with bacteria from various sources, including areas outside the pig farms. This could also explain the particularly high richness values found in the present study’s air samples. In contrast to the animal samples, we did not find farm-specific cecal microbiota among pig workers. Due to the similarity in life style and the narrow geographical region from where the human samples were received, we expect that the diet has a stronger influence on the farmers’ gut microbiota than the exposure to their individual farm. The farm specific part of the microbiota may therefore be too diluted to be detectable in such a diverse niche as the gut microbiota, whereas the nasal microbiota, seems to be strongly influenced by the exposure to its occupational environment as in a previous study a farm-specific nasal microbiota was observable in pig workers.^12^

The presence of animal-associated bacteria in the gut of pig workers may have some important implications. Farm environments are sources of antimicrobial resistance genes (ARG).^25^ In cases of the zoonotic transmission of bacteria, ARG could be co-transmitted. This might be particularly relevant for *E. coli*, which is well known for carrying the ESBL and *mcr* genes.^26–31^ *E. coli* transmission seems to occur, as shown by the Chinese study, but it was not found to be relevant in our setting.^22, Moor, submitted^ It is perhaps also relevant that we did not find any influence of antimicrobial treatments on the gut microbiota of pigs, which is a topic of controversy in the literature.^32,33^ It has to be taken into consideration that our study was not well powered and different antimicrobials were administered which prevents drawing a final conclusion concerning the influence of antimicrobial substances on the gut microbiota of pigs.

The present study had some limitations. Although the output of DADA2 results in exact ASVs allowing to disentangle the *Prevotellaceae* ASVs co-occurring in porcine and pig worker feces but which were not part of the gut microbiotas of cattle workers, the resolution is still restricted to a section of the 16S rRNA region. A more in-depth analysis like full-length 16S rRNA gene sequencing or shotgun metagenomics would have the potential to provide species up to strain-level taxonomic resolution.^34^ However, the some what high error rate of long-read sequencing platforms and the assembly of genomes of closely related bacterial species can also be challenging. As this was an observational study, no definitive conclusions about potentially health-related influences can be drawn.

The present study had some unique strengths. We simultaneously included samples from pigs (for three growth stages), air and pig workers. This One Health design allowed us to define which part of the airborne microbiota was likely transmissible from pigs to pig workers. In addition, a control group of cattle workers from the same geographical area was included. We speculated that the diet for the two different types of workers did not significantly differ and this, therefore, allowed the investigation of the influence of pig farming on the human gut microbiota in our study.

In conclusion, we demonstrated that occupational exposure to pigs altered the cecal microbiota of pig workers and that the duration of exposure modulated the intensity of this alteration. In particular, ASVs of *Prevotella* were commonly identified in the air, pigs, and pig workers’ cecal microbiota, indicating zoonotic transmission from animals to humans. The relevance of these findings for human health needs to be investigated in future studies. It will now be important to consider this finding when estimating occupational health risks, especially if the presence of pathogens and bacteria resistant to antimicrobials are suspected.

## Material and methods

### Sample collection

Prospective sampling was performed on pig workers and piglets selected from pig breeding farms in Switzerland. After their weaning period, the pigs were followed to their finishing units, located on either the same or a different farm. Pig farm workers self-collected stools and materials were sent to the laboratory by post. The request for human sampling was made at the first visit for workers on maternity units and on the single visit on fattening units, respectively. For both groups, samples did usually arrive within four weeks after the request; which corresponds to the late suckling; early weaning phase or late fattening stage, respectively. However, some unit workers had contact to pigs of varying age – between birth till slaughter (six months).

In parallel, rectal swabs from pigs were collected during their suckling (roughly two weeks old), weaning (six weeks old), and fattening (16 weeks old) periods using CultureSwab EZ. The time points were chosen due to the results of an earlier study which observed distinct shifts in microbiota structure along different growth stages.^24^ Care was taken to swab the identical animal three times, and this was achieved by using colored ear tags that ensured the retrieval of the right animals during subsequent visits. Air samples were collected from each barn, as previously described.^12,13^ Briefly, a sample of 3 m^3^ of air was collected from the central pen at roughly 1 m above the ground using a Coriolis device (Bertin technology, France). In addition, stools were obtained from a group of cattle workers of the same geographical area, who served as the study control group.

Ethical approval for this study was given by the Human Research Ethics Committee of the Canton of Vaud (2018–00080) and the Veterinary Ethics Committee of the Canton of Vaud (VD3335), both in Switzerland. Sample collection was conducted between October 2017 and March 2019 in the Swiss cantons of Vaud, Bern, Fribourg, and Jura.

### DNA extraction and 16S rRNA gene sequencing

After collection, rectal swabs were transported at 4°C and stored at −20°C until processing. Thawed swabs were suspended in 500 mL PBS using vortexing. DNA was extracted from the suspended swab and air samples following the QIAamp DNA Mini Kit spin protocol for the purification of genomic and viral DNA from body fluids. Aliquots of 200 mg of the self-collected fecal material samples from pig and cattle workers were stored at −80°C until processing using the QIAmp DNA Stool Mini Kit protocol. The V4 region of the 16S rRNA gene was amplified using forward (5ʹ-GTGCCAGCMGCCGCGGTAA-3ʹ) and reverse (5ʹ-GGACTACHVGGGTWTCTAAT-3ʹ) primers modified with an Illumina adaptor sequence at the 5ʹ end. PCR products were purified using the QIAquick PCR Purification Kit (Qiagen, Hilden, Germany). Samples were passed through to a MiSeq Illumina sequencing platform for indexing and paired-end sequencing (2 × 250 bp; reagent kit, v2).

### Sequencing analyses for the characterization of the microbiota

Sequencing data were analyzed as previously described using the DADA2 package (version 1.16.0) in R software (version 4.0.2) for the identification of amplicon sequence variants (ASV).^12,13^ DADA2 clusters the sequences into distinct ASVs^35^ which is in contrast to the operational taxonomic units (OTUs) output format generated by pipelines like *mothur*.^36^ A disadvantage of the DADA2 analysis was its potential overestimation of abundance due to duplications of the 16S rRNA genes within bacterial species. However, the pipeline’s ability to disentangle ASVs from OTUs which show more than 97% sequence similarity was a particular strength of the pipeline used in our study.

The taxonomy assignment of the ASVs was done using the SILVA (version 132) database. Contaminating sequences were identified using the *decontam* package (version 1.8.0) in R. Contaminants were identified by their frequency of occurrence and independently within each batch. Based on the ASVs identified using DADA2, we calculated the alpha-diversity values for richness and Shannon diversity indices (SD) using the *estimate_richness* command in the *phyloseq* package (version 1.32.0) in R. The alpha-diversity values for all the sample types (pig workers, cattle workers, air, suckling, weaning, and fattening pigs) were calculated and statistically analyzed using Wilcoxon rank-sum tests (using the *wilcox.test* function in R). Venn diagrams were drawn to illustrate the number of shared ASVs across the different sample types using the *vennDiagram* package (version 1.6.20). The corrected data sets were further analyzed using the *DESeq2* package (version 1.28.1) in R to make a differential analysis of ASVs of pig workers and cattle workers. The *p*-values were adjusted using Benjamini-Hochberg correction during the *DeSeq2* analysis. Results were represented in a volcano plot using the *lfcShrink* and *EnhancedVolcano* functions. The heatmap, displaying the relative abundance of ASVs among the different sample types (pig workers, cattle workers, air, suckling, weaning, and fattening pigs) was created using the *phytools* package (version 0.7.70) in R, and the phylogenetic tree was calculated using *webPRANK* (https://www.ebi.ac.uk/goldman-srv/webprank/).

Throughout this manuscript, we use two differently calculated distance matrices for our beta-diversity analyses: presence–absence Jaccard matrices and abundance-based Ružička matrices. Pairwise distances between samples were calculated using the *vegdist* function in R, and the resulting dissimilarity matrices were used to generate non-metric multidimensional scaling (NMDS) plots (the *metaMDS* function). Clustering in the different sampling types was analyzed statistically using 1,000 Monte Carlo permutation tests (PERMANOVA; the *adonis* function). Analyses of similarities were performed to investigate significant differences between different sample types using 1,000 Monte Carlo permutation tests (ANOSIM; the *anosim* function). The dissimilarity values were also used to calculate the within- and between-farm diversity indices among sample types. Results were represented using dissimilarity boxplots and analyzed statistically using Wilcoxon rank-sum tests (the *wilcox.test* function).

## Supplementary Material

Supplemental Material

## Accession number(s)

The sequencing reads for this study were deposited at the NCBI Sequence Read Archive under accession no. PRJEB42768.

## References

[1] Costea PI, Hildebrand F, Arumugam M, Backhed F, Blaser MJ, Bushman FD, De Vos WM, Ehrlich SD, Fraser CM, Hattori M, et al. Enterotypes in the landscape of gut microbial community composition. Nat Microbiol. 2018;3(1):8–13. doi:10.1038/s41564-017-0072-8. 29255284 PMC5832044

[2] David LA, Maurice CF, Carmody RN, Gootenberg DB, Button JE, Wolfe BE, Ling AV, Devlin AS, Varma Y, Fischbach MA, et al. Diet rapidly and reproducibly alters the human gut microbiome. Nature. 2014;505(7484):559–563. doi:10.1038/nature12820. 24336217 PMC3957428

[3] Yatsunenko T, Rey FE, Manary MJ, Trehan I, Dominguez-Bello MG, Contreras M, Magris M, Hidalgo G, Baldassano RN, Anokhin AP, et al. Human gut microbiome viewed across age and geography. Nature. 2012;486(7402):222–227. doi:10.1038/nature11053. 22699611 PMC3376388

[4] Wu GD, Chen J, Hoffmann C, Bittinger K, Chen YY, Keilbaugh SA, Bewtra M, Knights D, Walters WA, Knight R, et al. Linking long-term dietary patterns with gut microbial enterotypes. Science. 2011;334(6052):105–108. doi:10.1126/science.1208344. 21885731 PMC3368382

[5] Mulder M, Radjabzadeh D, Kiefte-de Jong JC, Uitterlinden AG, Kraaij R, Stricker BH, Verbon A. Long-term effects of antimicrobial drugs on the composition of the human gut microbiota. Gut Microbes. 2020;12(1):1795492. doi:10.1080/19490976.2020.1791677. PMC7781642 32991820

[6] Leo S, Lazarevic V, Gaia N, Estellat C, Girard M, Matheron S, Armand-Lefevre L, Andremont ATV-R, Schrenzel J, Ruppe E. The intestinal microbiota predisposes to traveler’s diarrhea and to the carriage of multidrug-resistant Enterobacteriaceae after traveling to tropical regions. Gut Microbes. 2019;10(5):631–641. doi:10.1080/19490976.2018.1564431. 30714464 PMC6748584

[7] Pires J, Kraemer JG, Kuenzli E, Kasraian S, Tinguely R, Hatz C, Endimiani A, Hilty M. Gut microbiota dynamics in travelers returning from India colonized with extended-spectrum cephalosporin-resistant Enterobacteriaceae: a longitudinal study. Travel Med Infect Dis. 2019;27:72–80. doi:10.1016/j.tmaid.2018.10.012. 30339827

[8] Pietroiusti A, Bergamaschi E, Campagna M, Campagnolo L, De Palma G, Iavicoli S, Leso V, Magrini A, Miragoli M, Pedata P, et al. The unrecognized occupational relevance of the interaction between engineered nanomaterials and the gastro-intestinal tract: a consensus paper from a multidisciplinary working group. Part Fibre Toxicol. 2017;14(1):47. doi:10.1186/s12989-017-0226-0. 29178961 PMC5702111

[9] Zhou Y, Chen L, Sun G, Li Y, Huang R. Alterations in the gut microbiota of patients with silica-induced pulmonary fibrosis. J Occup Med Toxicol. 2019;14(1):5. doi:10.1186/s12995-019-0225-1. 30867671 PMC6399897

[10] Patterson AD, Turnbaugh PJ. Microbial determinants of biochemical individuality and their impact on toxicology and pharmacology. Cell Metab. 2014;20(5):761–768. doi:10.1016/j.cmet.2014.07.002. 25156450 PMC4252706

[11] Claus SP, Guillou H, Ellero-Simatos S. The gut microbiota: a major player in the toxicity of environmental pollutants? NPJ Biofilms Microbiomes. 2016;2(1):16003. doi:10.1038/npjbiofilms.2016.3. 28721242 PMC5515271

[12] Kraemer JG, Ramette A, Aebi S, Oppliger A, Hilty M. Influence of pig farming on the human’s nasal microbiota: the key role of the airborne microbial communities. Appl Environ Microbiol. 2018;84(6). doi:10.1128/AEM.02470-17. PMC5835734 29330190

[13] Kraemer JG, Aebi S, Oppliger A, Hilty M. The indoor-air microbiota of pig farms drives the composition of the pig farmers’ nasal microbiota in a season-dependent and farm-specific manner. Appl Environ Microbiol. 2019;85(9):9. doi:10.1128/AEM.03038-18. PMC6495764 30824439

[14] Tan SC, Chong CW, Yap IKS, Thong KL, Teh CSJ. Comparative assessment of faecal microbial composition and metabonome of swine, farmers and human control. Sci Rep. 2020;10(1):8997. doi:10.1038/s41598-020-65891-4. 32488118 PMC7265441

[15] Amat S, Lantz H, Munyaka PM, Willing BP. *Prevotella* in pigs: the positive and negative associations with production and health. Microorganisms. 2020;8(10):10. doi:10.3390/microorganisms8101584. PMC7602465 33066697

[16] Ley RE. Gut microbiota in 2015: prevotella in the gut: choose carefully. Nat Rev Gastroenterol Hepatol. 2016;13(2):69–70. doi:10.1038/nrgastro.2016.4. 26828918

[17] Brooks JP, McLaughlin MR, Gerba CP, Pepper IL. Land application of manure and Class B biosolids: an occupational and public quantitative microbial risk assessment. J Environ Qual. 2012;41(6):2009–2023. doi:10.2134/jeq2011.0430. 23128758

[18] Tanner BD, Brooks JP, Gerba CP, Haas CN, Josephson KL, Pepper IL. Estimated occupational risk from bioaerosols generated during land application of class B biosolids. J Environ Qual. 2008;37(6):2311–2321. doi:10.2134/jeq2007.0193. 18948485

[19] De Matos Nascimento A, De Paula VR, Dias EHO, Da Costa Carneiro J, Otenio MH. Quantitative microbial risk assessment of occupational and public risks associated with bioaerosols generated during the application of dairy cattle wastewater as biofertilizer. Sci Total Environ. 2020;745:140711. doi:10.1016/j.scitotenv.2020.140711. 32763641

[20] Burch TR, Spencer SK, Stokdyk JP, Kieke BA Jr., Larson RA, Firnstahl AD, Rule AM, Borchardt MA. Quantitative microbial risk assessment for spray irrigation of dairy manure based on an empirical fate and transport model. Environ Health Perspect. 2017;125(8):087009. doi:10.1289/EHP283. 28885976 PMC5884668

[21] Masclaux FG, Sakwinska O, Charriere N, Semaani E, Oppliger A. Concentration of airborne *Staphylococcus aureus* (MRSA and MSSA), total bacteria, and endotoxins in pig farms. Ann Occup Hyg. 2013;57(5):550–557. doi:10.1093/annhyg/mes098. 23293050

[22] Sun J, Liao XP, D’Souza AW, Boolchandani M, Li SH, Cheng K, Luis Martinez J, Li L, Feng YJ, Fang LX, et al. Environmental remodeling of human gut microbiota and antibiotic resistome in livestock farms. Nat Commun. 2020;11(1):1427. doi:10.1038/s41467-020-15222-y. 32188862 PMC7080799

[23] Bergamaschi M, Tiezzi F, Howard J, Huang YJ, Gray KA, Schillebeeckx C, McNulty NP, Maltecca C. Gut microbiome composition differences among breeds impact feed efficiency in swine. Microbiome. 2020;8(1):110. doi:10.1186/s40168-020-00888-9. 32698902 PMC7376719

[24] Wang X, Tsai T, Deng F, Wei X, Chai J, Knapp J, Apple J, Maxwell CV, Lee JA, Li Y, et al. Longitudinal investigation of the swine gut microbiome from birth to market reveals stage and growth performance associated bacteria. Microbiome. 2019;7(1):109. doi:10.1186/s40168-019-0721-7. 31362781 PMC6664762

[25] Munk P, Knudsen BE, Lukjancenko O, Duarte ASR, Van Gompel L, Luiken REC, Smit LAM, Schmitt H, Garcia AD, Hansen RB, et al. Abundance and diversity of the faecal resistome in slaughter pigs and broilers in nine European countries. Nat Microbiol. 2018;3(8):898–908. doi:10.1038/s41564-018-0192-9. 30038308

[26] Seiffert SN, Hilty M, Perreten V, Endimiani A. Extended-spectrum cephalosporin-resistant Gram-negative organisms in livestock: an emerging problem for human health? Drug Resist Updat. 2013;16:22–45. 23395305 10.1016/j.drup.2012.12.001

[27] Rhouma M, Beaudry F, Theriault W, Letellier A. Colistin in pig production: chemistry, mechanism of antibacterial action, microbial resistance emergence, and one health perspectives. Front Microbiol. 2016;7:1789. doi:10.3389/fmicb.2016.01789. 27891118 PMC5104958

[28] De Been M, Lanza VF, De Toro M, Scharringa J, Dohmen W, Du Y, Hu J, Lei Y, Li N, Tooming-Klunderud A, et al. Dissemination of cephalosporin resistance genes between *Escherichia coli* strains from farm animals and humans by specific plasmid lineages. PLoS Genet. 2014;10(12):e1004776. doi:10.1371/journal.pgen.1004776. 25522320 PMC4270446

[29] Dohmen W, Bonten MJ, Bos ME, Van Marm S, Scharringa J, Wagenaar JA, Heederik DJ. Carriage of extended-spectrum beta-lactamases in pig farmers is associated with occurrence in pigs. Clin Microbiol Infect. 2015;21(10):917–923. doi:10.1016/j.cmi.2015.05.032. 26033669

[30] Dohmen W, Dorado-Garcia A, Bonten MJ, Wagenaar JA, Mevius D, Heederik DJ. Risk factors for ESBL-producing *Escherichia coli* on pig farms: a longitudinal study in the context of reduced use of antimicrobials. PLoS One. 2017;12(3):e0174094. doi:10.1371/journal.pone.0174094. 28323856 PMC5360262

[31] Dohmen W, Schmitt H, Bonten M, Heederik D. Air exposure as a possible route for ESBL in pig farmers. Environ Res. 2017;155:359–364. doi:10.1016/j.envres.2017.03.002. 28273621

[32] Looft T, Johnson TA, Allen HK, Bayles DO, Alt DP, Stedtfeld RD, Sul WJ, Stedtfeld TM, Chai B, Cole JR, et al. In-feed antibiotic effects on the swine intestinal microbiome. Proc Natl Acad Sci U S A. 2012;109(5):1691–1696. doi:10.1073/pnas.1120238109. 22307632 PMC3277147

[33] Mencia-Ares O, Cabrera-Rubio R, Cobo-Diaz JF, Alvarez-Ordonez A, Gomez-Garcia M, Puente H, Cotter PD, Crispie F, Carvajal A, Rubio P, et al. Antimicrobial use and production system shape the fecal, environmental, and slurry resistomes of pig farms. Microbiome. 2020;8(1):164. doi:10.1186/s40168-020-00941-7. 33213522 PMC7678069

[34] Johnson JS, Spakowicz DJ, Hong BY, Petersen LM, Demkowicz P, Chen L, Leopold SR, Hanson BM, Agresta HO, Gerstein M, et al. Evaluation of 16S rRNA gene sequencing for species and strain-level microbiome analysis. Nat Commun. 2019;10(1):5029. doi:10.1038/s41467-019-13036-1. 31695033 PMC6834636

[35] Callahan BJ, McMurdie PJ, Rosen MJ, Han AW, Johnson AJ, Holmes SP. DADA2: high-resolution sample inference from Illumina amplicon data. Nat Methods. 2016;13(7):581–583. doi:10.1038/nmeth.3869. 27214047 PMC4927377

[36] Schloss PD. Reintroducing mothur: 10 Years Later. Appl Environ Microbiol. 2020;86:2. 10.1128/AEM.02343-19 PMC6952234 31704678

