# Supplementary material for: Influence of pig farming on human Gut Microbiota: role of airborne microbial communities
Source: Gut Microbes. 2021 Jun 1;13(1):1927634. doi: 10.1080/19490976.2021.1927634 (PMC8172160; doi:10.1080/19490976.2021.1927634)

Supplementary figure 1: Heat map of the relative abundances of ASVs associated with pig farming

A total of 159 ASVs were significantly associated with either working on a cattle or pig farm (see main text for details). The phylogenetic tree is based on differences in ASV sequenced reads (left half). The heat map shows the relative abundance of ASVs for pigs, pig workers (Pw), cattle workers (Cw), and air. The taxonomic assignment of the ASVs with high relative abundances are indicated in the figure (right half). Standardized values were created using the *phylo.heatmap* function (each column was standardized to have the same variance and mean prior to analysis) enabling the identification of small differences in relative abundance.

Supplementary figure 1; Heat map

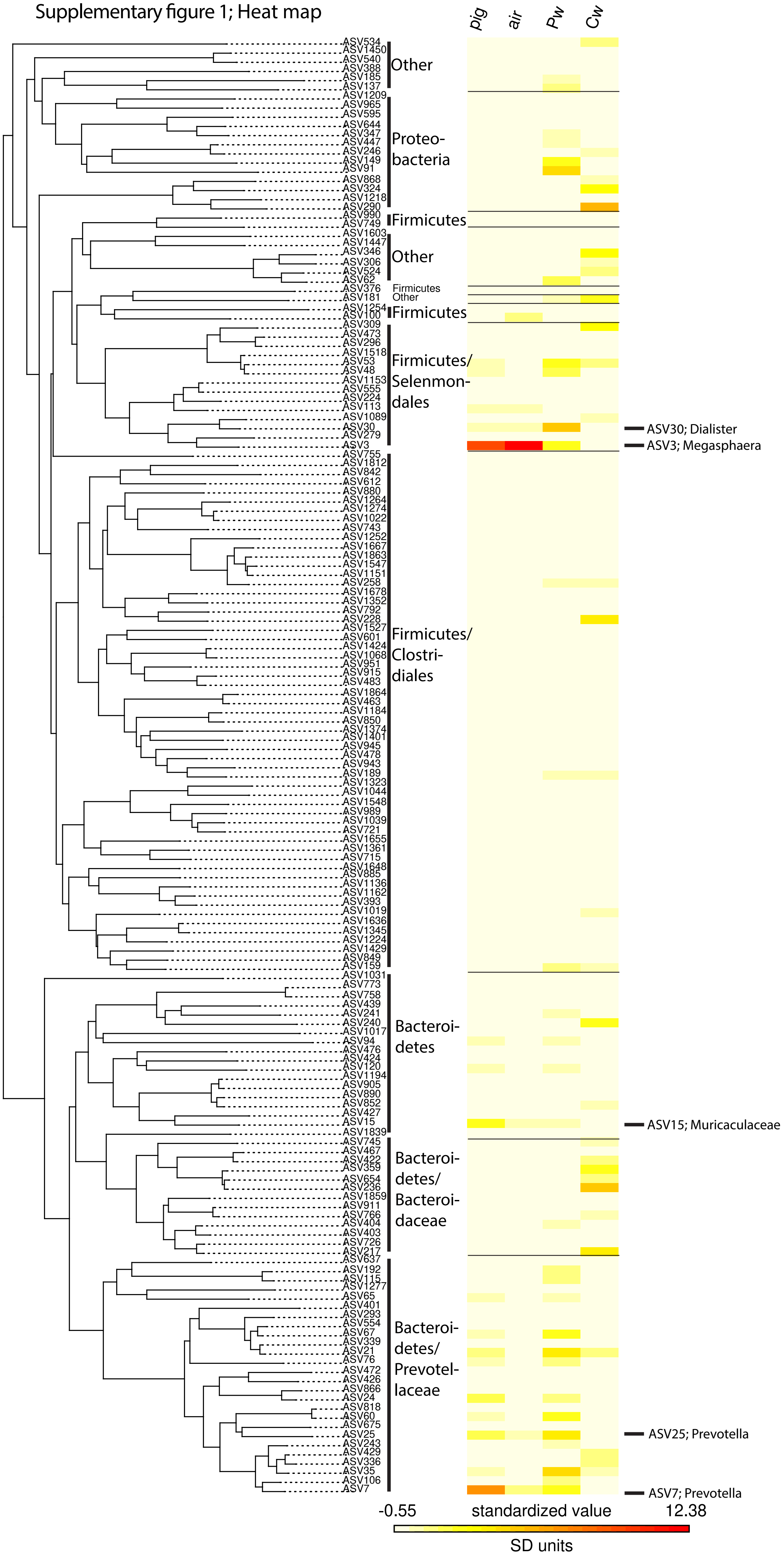

Supplementary figure 2: Within- and between-pig-farm dissimilarity analyses. (A) shows unweighted (Jaccard) and (B) shows weighted (Ružička) distance matrices values for 'within-farm' and 'between-farm' microbiota composition. Dissimilarity index values of pig workers to pigs at the three growth stages (suckling (suck), weaning (wean), fattening (fat) and to air showed no significant within-between farm diversity effect.

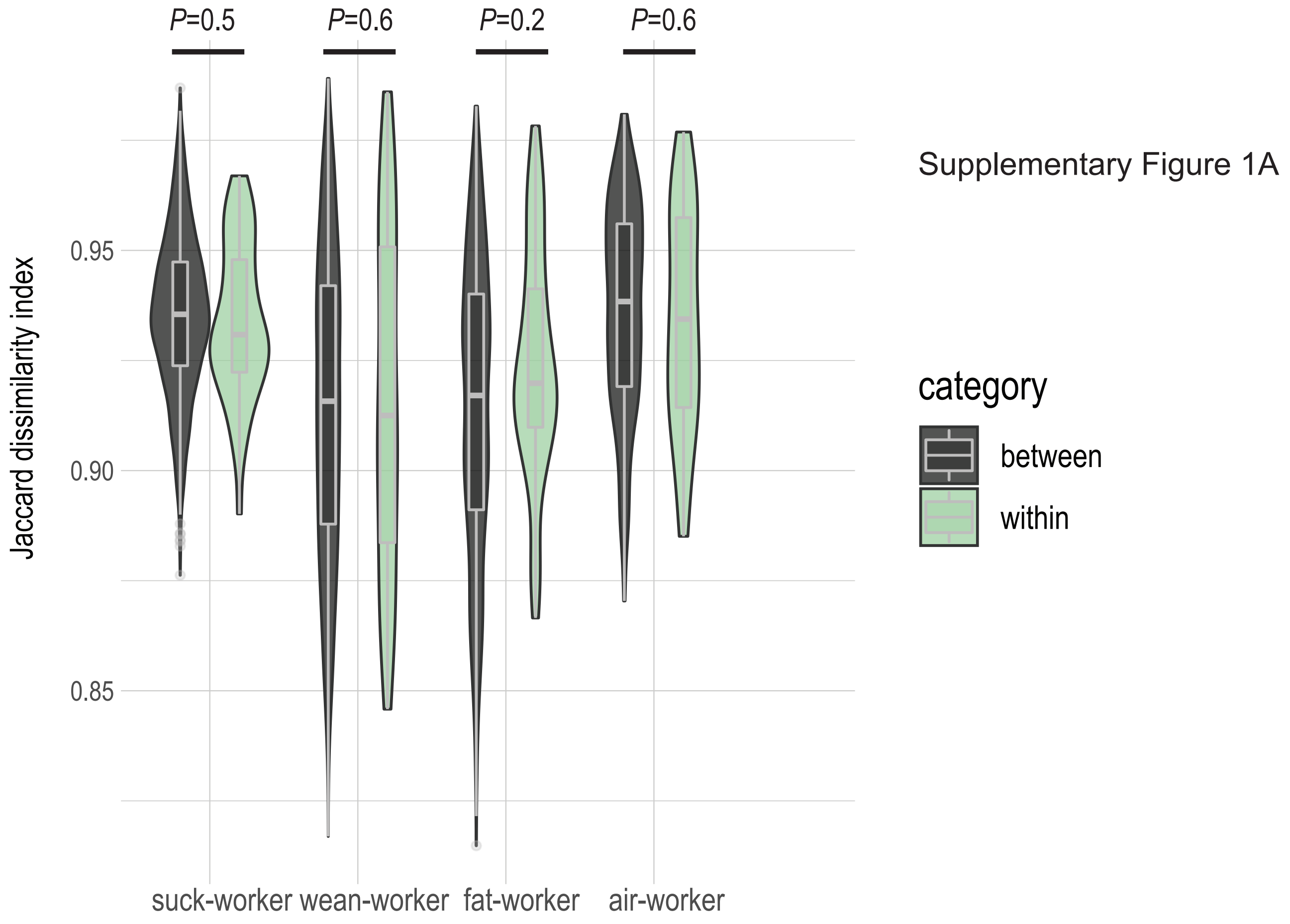

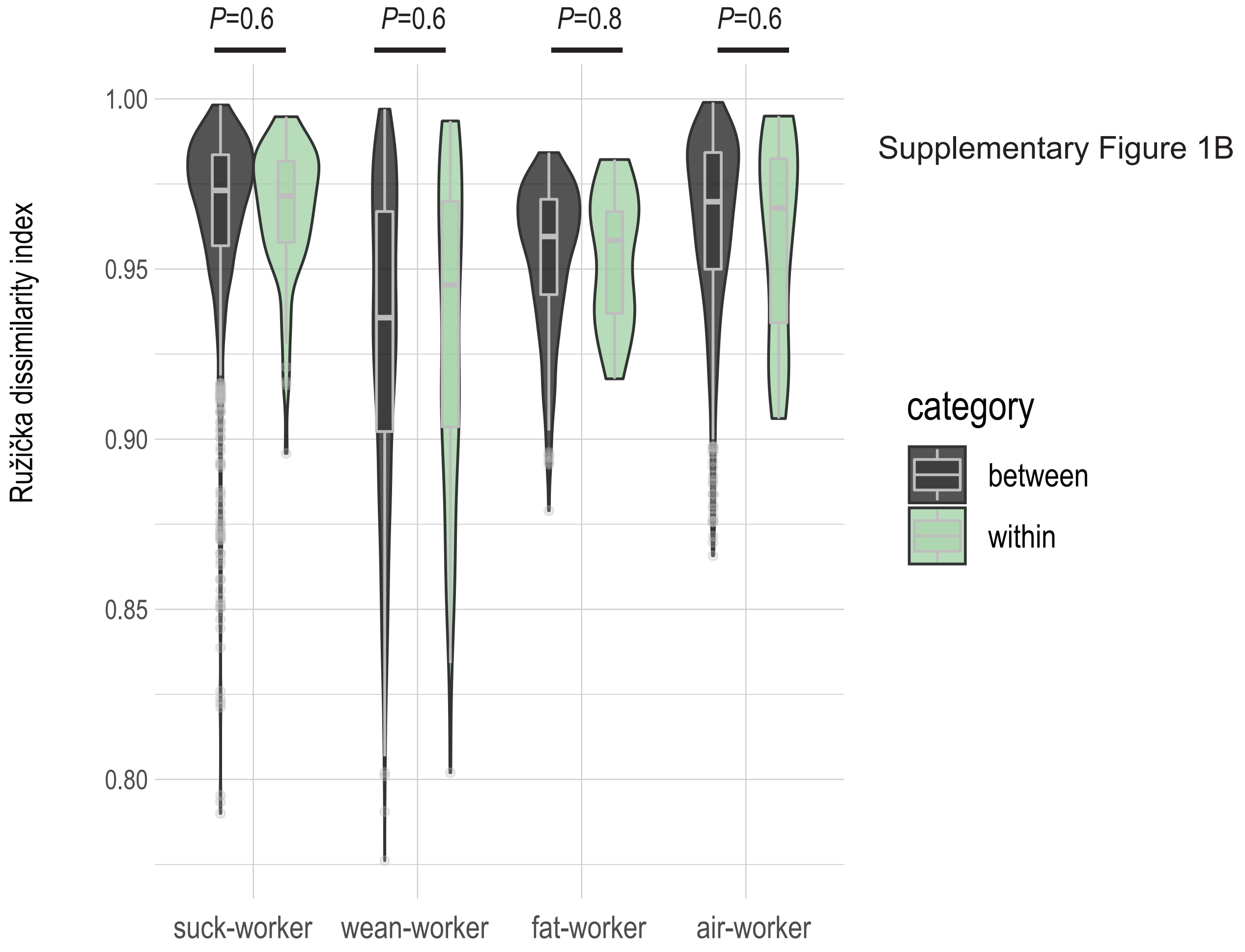

Supplement: Supplemental Material [file KGMI_A_1927634_SM6717.zip › Supplementary information/Figures_S1_and_S2.pdf]
